# Elimination of trachoma as a public health problem in Ghana: Providing evidence through a pre-validation survey

**DOI:** 10.1371/journal.pntd.0006099

**Published:** 2017-12-12

**Authors:** Oscar Debrah, Ernest O. Mensah, Laura Senyonjo, Dziedzom K. de Souza, Tei E. Hervie, David Agyemang, Didier Bakajika, Benjamin Marfo, Felix Ahorsu, Seth Wanye, Robin Bailey, Joseph B. Koroma, Agatha Aboe, Nana-Kwadwo Biritwum

**Affiliations:** 1 Eye Care, Ghana Health Service, Accra, Ghana; 2 FHI 360, Ghana office, Accra, Ghana; 3 Sightsavers International, UK Office, London, United Kingdom; 4 London School of Hygiene and Tropical Medicine, London, United Kingdom; 5 Noguchi Memorial Institute for Medical Research, University of Ghana, Accra, Ghana; 6 Neglected Tropical Diseases Program, Ghana Health Service, Accra, Ghana; 7 Sightsavers International, Ghana Office, Accra, Ghana; RTI International, UNITED STATES

## Abstract

**Background:**

In order to achieve elimination of trachoma, a country needs to demonstrate that the elimination prevalence thresholds have been achieved and then sustained for at least a two-year period. Ghana achieved the thresholds in 2008, and since 2011 has been implementing its trachoma surveillance strategy, which includes community and school screening for signs of follicular trachoma and trichiasis, in trachoma-endemic districts. In 2015–2016, the country conducted a district level population-based survey to validate elimination of trachoma as a public health problem.

**Methods:**

As per WHO recommendations, a cross-sectional survey, employing a two-stage cluster random sampling methodology, was used across 18 previously trachoma endemic districts (evaluation units (EUs) in the Upper West and Northern Regions of Ghana. In each EU 24 villages were selected based on probability proportional to estimated size. A minimum of 40 households were targeted per village and all eligible residents were examined for clinical signs of trachoma, using the WHO simplified grading system. The number of trichiasis cases unknown to the health system was determined. Household environmental risk factors for trachoma were also assessed.

**Results:**

Data from 45,660 individuals were examined from 11,099 households across 18 EUs, with 27,398 (60.0%) children aged 1–9 years and 16,610 (36.4%) individuals 15 years and above All EUs had shown to have maintained the WHO elimination threshold for Trachomatous inflammation-Follicular (TF) (<5.0% prevalence) in children aged 1–9 years old. The EU TF prevalence in children aged 1–9 years old ranged from between 0.09% to 1.20%. Only one EU (Yendi 0.36%; 95% CI: 0.0–1.01) failed to meet the WHO TT elimination threshold (< 0.2% prevalence in adults aged 15 and above). The EU prevalence of trichiasis (TT) unknown to the health system in adults aged ≥15 years, ranged from 0.00% to 0.36%. In this EU, the estimated TT backlog is 417 All TT patients identified in the study, as well as through on-going surveillance efforts will require further management. A total of 75.9% (95% CI 72.1–79.3, EU range 29.1–92.6) of households defecated in the open but many households had access to an improved water source 75.9% (95%CI: 71.5–79.8, EU range 47.4–90.1%), with 45.5% (95% CI 41.5–49.7%, EU range 28.4–61.8%) making a round trip of water collection < 30 minutes.

**Conclusion:**

The findings from this survey indicate elimination thresholds have been maintained in Ghana in 17 of the 18 surveyed EUs. Only one EU, Yendi, did not achieve the TT elimination threshold. A scheduled house-by-house TT case search in this EU coupled with surgery to clear the backlog of cases is necessary in order for Ghana to request validation of elimination of trachoma as a public health problem.

## Author summary

Trachoma is an eye disease caused by an infection with *Chlamydia trachomatis*. It is the leading cause of blindness due to infection globally. However, trachoma is both treatable and preventable through antibiotics and surgery, and may be preventable with basic hygiene improvements. Thus, the Surgery, Antibiotics, Facial cleanliness and Environmental modification (SAFE) strategy is endorsed by the World Health Organization for the implementation of interventions. Trachoma is a progressive disease. Clinically active trachoma, the follicular (trachomatous-inflammation follicular (TF)) and intense (trachomatous- inflammation intense (TI)) phases are most common in children aged 1–9 years old while complications such as in turned lashes and loss of vision are found mainly in adults. Globally, the disease is earmarked for elimination as a public health problem by the year 2020. In Ghana, studies conducted in 2000–2003 identified trachoma as endemic in the Upper West and Northern regions and by 2004 the elimination strategy based on Surgery, Antibiotics treatment, Face washing and Environmental improvement, was being implemented in all 18 districts in the two regions. Surveys conducted in 2008 indicated that the strategy had been successful with reduction of clinically active trachoma to elimination levels in all 18 districts. Ghana implemented a post-elimination surveillance phase from 2011–2014 and conducted a formal prevalence survey in 2015–2016 using WHO standard grading methodologies, the results of which are presented here. The results showed that TF prevalence was below the 5% elimination target in children aged 1–9 years old in all 18 districts, while TT was below 2 cases in every 1000 persons aged 15 and above in all 18 endemic districts except one (Yendi district). Ghana has put in place a TT case search of all persons 15 years and above in the Yendi district to identify persons with the complications and offer surgery to them. The outcomes of these investigations will enable Ghana to provide evidence for WHO dossier submission, to acknowledge validation of elimination of trachoma as public health problem.

## Introduction

Trachoma, a result of infection with the bacterium C*hlamydia trachomatis*, remains the leading infectious cause of blindness worldwide [1]. The intervention strategy for trachoma is the World Health Organization (WHO)-endorsed SAFE strategy (S: Surgery for in-turned eyelashes as a result of trachomatous trichiasis; A: Antibiotics to clear *Chlamydia trachomatis* infection; F: Facial cleanliness and E: Environmental improvement to reduce transmission of *C*. *trachomatis*) [2]. Based on the implementation and scale-up of this strategy, the World Health Assembly set the global goal to eliminate trachoma by 2020 [3].

Countries are eligible for acknowledgment of elimination of trachoma when they provide evidence that all endemic districts (Evaluation Units–EUs) have reached the elimination prevalence thresholds of <5.0% for trachomatous inflammation-follicular (TF) in children 1–9-years old, and <0.2% for trachomatous trichiasis (TT) in individuals 15 years and above and sustained those achievements for at least two years [4]. To evidence this and ensure there is no re-emergence of infection, WHO recommends a population-based prevalence survey to be implemented at least two years after achieving the elimination thresholds.

The earliest published reports of trachoma in Ghana, which identified trachoma as a major cause of blindness, were by Sarkies [5] in 1952 and Rodger [6] in 1959 in Northern Ghana. The 1993 Annual Report of the Bawku Presbyterian Rural Eye Programme, which was based in the Upper East Region of Ghana but extended their services to the Northern and Upper West Regions, highlighted trachoma as a problem in the Northern and Upper West Regions. A review of data from eye clinics in the country at the then Eye Care Secretariat (now National Eye Health Unit) showed a high proportion of conjunctivitis in the Northern and Upper West Region which informed a decision to have a detailed look at the two regions. A rapid assessment of trachoma was done in 1997 in the Daboya sub-district of the Northern Region. The results showed the presence of trachoma in the sub-district. In December 1998, the Ministry of Health, WHO and Non-Governmental Development Organizations (NGDO) collaborated in an effort to assess the trachoma situation in the two-suspected trachoma endemic regions, and ranked the affected villages for prioritizing trachoma control interventions. A modified version of the WHO Trachoma Rapid Assessment (TRA3) methodology [7] was used to prioritize all the 18 districts in the Northern (13 districts) and Upper West (5 districts) regions for intervention. The results showed that approximately 70% of the 122 villages assessed, in both regions were endemic for trachoma, and thus the districts would need interventions using the various components of the WHO-endorsed “SAFE” strategy [2] for the elimination of trachoma.

In 1999–2000, the programme conducted population-based baseline epidemiological trachoma prevalence surveys in areas suspected to be endemic for the disease [8]. Initially, five districts were surveyed in 2000; all five districts (Tolon-Kumbungu, Savelugu-Nanton and Tamale in the Northern region and Wa and Sissala in the Upper West region), had trachoma of public health significance needing interventions with the various components of the SAFE strategy. Ghana was among the first sixteen countries recommended by the World Health Organization (WHO) for the elimination of blinding trachoma. The International Trachoma Initiative (ITI) initially selected Ghana, alongside five other countries, for the elimination of trachoma as a public health problem. The Ghana National Trachoma Control Programme was initiated in June 2000 by the Ghana Health Service (GHS)/ Ministry of Health (MOH) together with its partners (International Trachoma Initiative (ITI), The Carter Center, CBM, Sightsavers, Swiss Red Cross and WHO) to eliminate trachoma as a public health problem in the Northern and Upper West Regions. The WHO-endorsed SAFE strategy [2] using the Pfizer-donated Zithromax was started in all known endemic communities in the initial five surveyed districts by 2001 [9]. A population-based baseline epidemiological trachoma prevalence survey conducted in a sixth district (West Gonja in the Northern region) in 2002, and the SAFE strategy extended to this district. All known endemic communities in this district were added to the programme. An evaluation survey in the first five districts after two years of implementing control activities showed between 41–79% reduction in the prevalence of active trachoma [10,11]. In 2003, the programme conducted baseline surveys in the remaining 12 of the 18 districts in the two endemic regions. All the districts showed various levels of endemicity of trachoma and were all added to the programme, by 2004.

The Ghana programme developed National Plans to guide programme implementation. In 2000, the first two-year plan for the implementation of the SAFE strategy was developed. In 2003, the first strategic plan (Programme Information and Planning Document, 2003–2007) was developed and launched to guide trachoma control in Ghana. In 2005, it became necessary to develop a new national strategic document as more districts were being brought on board and new guidelines from WHO [2] were introduced. With a full knowledge of trachoma-endemic areas, the National Trachoma Control Program developed a five-year strategic plan (2005–2009) to guide trachoma control activities [12]. The plan set various targets with the ultimate goal to eliminate trachoma from Ghana by 2010. The plan also provided benchmarks to assess programme performance, such as evaluating the impact of three years of SAFE strategy on the prevalence of disease.

Ghana is one of the countries in the pre-validation surveillance stage [13], having initially achieved the elimination thresholds but not yet having conducted the validation surveys to provide evidence that elimination thresholds have been maintained. Following an impact assessment in 2008 after all endemic communities and districts had received at least three years of SAFE interventions, the prevalence of TF fell to 0.1–2.9% from a baseline of 2.8–16.1% in 2003 [14]. These results revealed that Ghana had reduced TF prevalence in children aged 1–9 years to less than 5.0% in all endemic districts. However, the current criteria for validation of elimination of trachoma requires that TF in children aged 1–9 years remains at <5.0% for at least two years after interventions to control trachoma have ceased [15,16]. Further, the target threshold for the elimination of trichiasis is a prevalence of TT <0.2% in 15 years and older [17]. TT prevalence ranges fell from the baseline figures of 0.4%-8.4% in 2003 to 0.1–1.1% in 2008 [14] but was still above the recommended threshold for validation of elimination in some districts (S1 Table).

Following the impact assessment, a surveillance strategy based on WHO recommendations at the time, was put in place between 2011 and 2014. This involved both active and passive surveillance for TF and TT cases. WHO recommendations require Ghana to conduct final EU level trachoma prevalence surveys to validate that the country has maintained the elimination thresholds eliminated trachoma as a public health problem in the country. This study is one of the first EU-level pre-validation surveys using the new WHO guidelines.

## Methods

The publication of this study follows the STROBE (STrengthening the Reporting of OBservational studies in Epidemiology) checklist to document its compliance with STROBE guidelines (S1 Checklist).

### Ethical considerations

The study was reviewed by the Ghana Health Service Ethics Review Committee (GHS-ERC 03/07/15) in Ghana, and the London School of Hygiene and Tropical Medicine review board (Reference: 10285). Information about the study and the use of data was explained to study participants before they consented to taking part. Where they are unable to sign their name, a thumbprint was taken. The written informed consent of the household head and each study participant was obtained before data collection and specimen collection. Parental consent was sought for children to participate in the study. Children also gave assent for the study. All individuals identified with TF were given azithromycin for treatment and TT cases referred to the health system for further management. All information collected was confidential and stored anonymously. All electronic databases were password-protected with logged, individual-level access only available to key staff.

### Study design

The study is a cross-sectional population-based survey, following a two-stage cluster sampling methodology, based on GTMP protocols, to validate if elimination targets have been sustained [18]. An assessment of clinical signs of trachoma was undertaken by trained ophthalmic nurses in December 2015 –February 2016.

### Study sites

**Fig 1:**
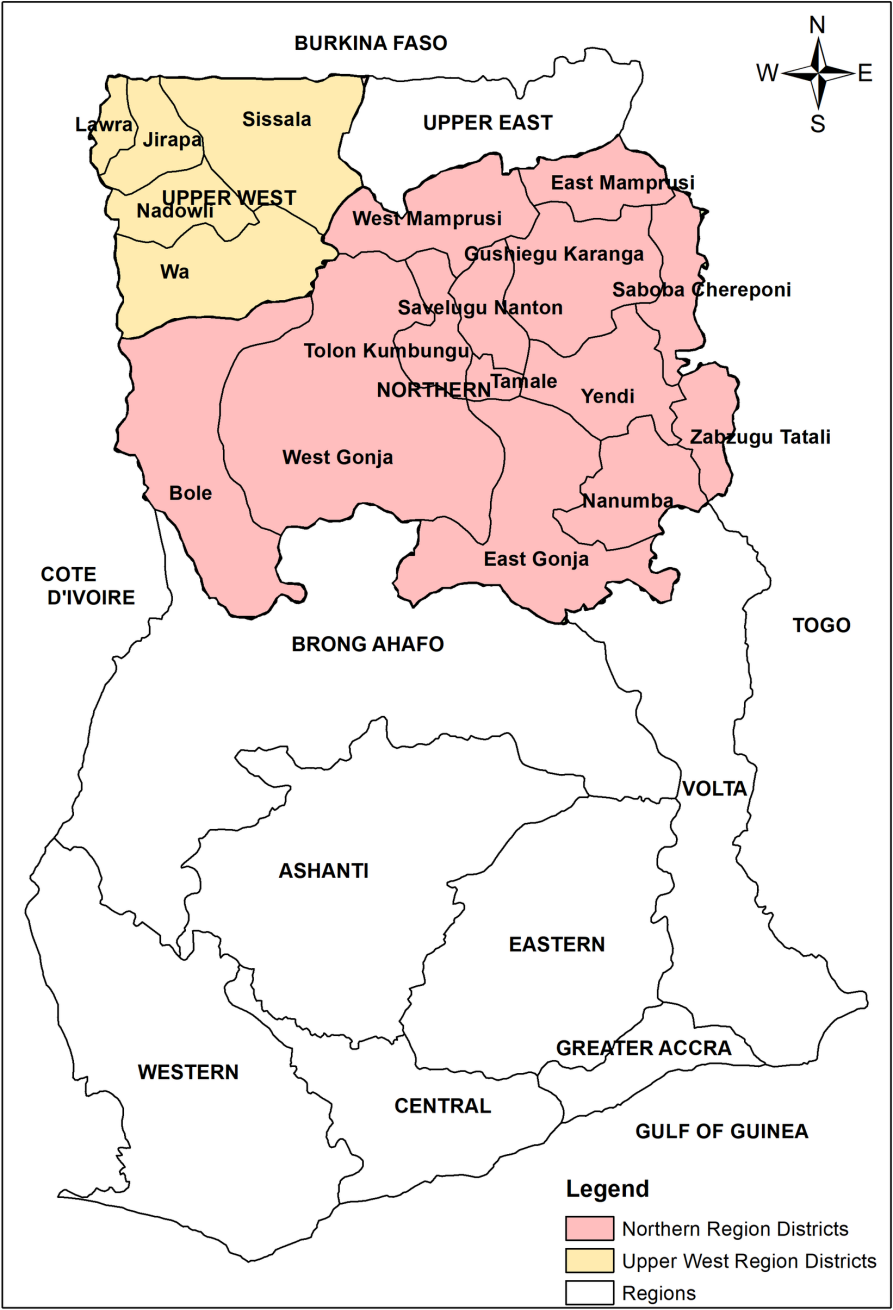
Map of Ghana, with survey regions and districts.

The study was conducted in the two trachoma-endemic regions of Northern and Upper West regions of Ghana (Fig 1). These regions experience a single rainy season, from May to October, when humidity generally reaches 70–90%. During the long dry season (November to April), the humidity drops significantly and the colder, dusty and dry harmattan winds blow northeast. A number of rivers run through the regions, including the tributaries of the Volta (Black Volta, White Volta), Kulpawn and Sissili. However, many communities in these regions rely on underground water, especially during the dry season. The literacy rate among adults in this part of the country is among the lowest in Ghana. The main economic activity in the regions is farming [19].

These two regions were identified as trachoma-endemic regions between 2000 and 2003 (see S1 Table). In 2008, all 18 EUs were declared to have achieved the elimination prevalence thresholds for TF and mass distribution of antibiotics was stopped.

For the purposes of this survey, districts were combined to their original parent districts (same as those used for the baseline study) to create 18 EUs with a population of between 100,000 to 250,000 inhabitants.

### Sample size

The sample size was calculated for the required precision of TF in children aged 1–9 years old, based on the following parameters: prevalence (p) of 4%, a precision (d) of +/- 2%, a Z value (z) of 1.96 or 95% confidence level, a design effect of 3.3 estimated from previous surveys, and an expected non-response rate of 10%.

Thus, a total of 1,338 children were required to be recruited for the study in each EU. This gives a total of 24,084 across the 18 EUs. Based on 2010 census, there is assumed to be an average of 1.7 children per household. Based on a total of 24 clusters per EU, a total of 33 households were to be sampled per cluster.

### Sample selection

The primary sampling unit, the village, was selected based on probability proportional to size. The secondary sampling unit, the household, was randomly selected using compact segment sampling [20,21].

All eligible individuals (i.e. individuals above the age of 1 year) in the household were sampled. Selected households were not replaced when residents were absent or refused examination. To minimize the number of residents missed in selected households, survey teams re-visited the household before leaving the village on the day of the survey.

### Survey data collection

The survey team was made up of one data recorder and one grader. The head of each selected household provided basic demographic information for each resident. Key Water Sanitation and Hygiene (WASH) indicators as outlined under the GTMP survey protocol, were also collected through the household interviews and through observation by the trained data recorders. GPS data for each household was collected. All individuals in the household were assessed for clinical signs of trachoma using the WHO simplified grading system. Facial cleanliness in terms of ocular and nasal discharge was also assessed in children aged 1–9 years old.

### Data management considerations and statistical analysis

Questionnaire data and clinical examination results were collected, using the LINKS system [22], a smartphone application allowing data to be entered electronically and sent to a centralized database server through an encrypted connection. Training was held on the use of the system in advance of field work to ensure correct and uniform usage of the tool. Information collected using the electronic data collection system was uploaded to a cloud server with password-protected access to the database only granted to collaborative study investigators for analysis.

Results from clinical examination were analysed to determine overall EU level adjusted cluster summarised means. TF prevalence data in children aged 1–9 years old was adjusted for age based on one-year age groups (using the 2010 Ghana census), and TT prevalence data in adults aged 15 and above was adjusted for sex and age in five-year age groups. As clusters were selected using probability proportional to size sampling, the data was assumed to be self-weighted. However, the analysis was adjusted to take into account the cluster sampling methodology used [23,24]. Confidence intervals around prevalence estimates were calculated by bootstrapping the adjusted cluster prevalence and taking the 2.5^th^ and 97.5^th^ centiles based on 10,000 iterations.

TT unknown to the health system was defined as an individual with TT and presence of trachomatous scarring (TS), who had not been offered surgery or other management and were not a recurrent case.

The WHO/UNICEF Joint Monitoring Programme standards were used to categorise relevant WASH variables. As such an “improved” sanitation facility was a private facility that hygienically separated excreta from human contact whilst an “improved” water source was one that is constructed to protect the source from outside contamination. [25,26]

The number of unknown TT patients was calculated as the sum of the district backlogs, where each district backlog was the product of the total population and the population prevalence of TT for that district.

Statistical analysis was undertaken using STATA 12 (College Station, TX: USA). Maps were drawn using ArcGIS 10.2.

### Quality assurance

#### Grader qualifying workshop

All trachoma graders were trained on the clinical diagnosis of trachoma. Before inclusion in the team, each grader had to achieve a kappa score of at least 0.7 (based on field grades) in an inter-grader agreement exercise with an experienced GTMP master grader trainer. The exercise was conducted with real-life examples of TF and due to the low prevalence of cases in Ghana, the training of graders was conducted in Sokoto, Nigeria, a location with a higher prevalence of trachoma.

#### Training for field activities

Before the start of the data collection, the trachoma graders and data recorders were trained over a one-week period, including both theory and practical sessions. The training covered issues of data collection, recording, sampling procedures and ethical considerations and pre-testing of the study protocols. Three ophthalmologists with experience in trachoma control were present during the training, pre-testing and supervision of actual data collection.

## Results

### Population demographics and household characteristics

**Fig 2:**
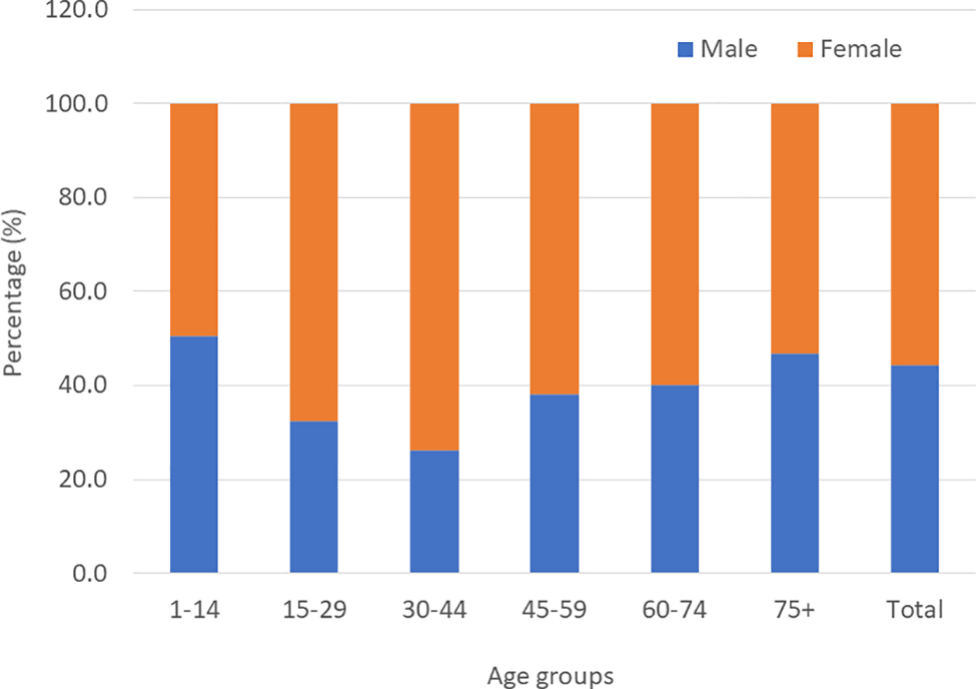
Age and sex distribution in the study population.

A total of 45,660 individuals consented for the study and provided responses, including 27,398 (60.0%) children aged 1–9 years and 16,610 (36.4%) individuals 15 years and above. 1652 (3.6%) participants’ data were not included in the determination of the prevalence estimates. These include individuals who refused to be examined (especially children), and adults who were registered as members of a household but who had not returned at the time of the mop-up visit by the survey team. The mean age of the study participants was 17 years. Overall, 44.3% were males and 55.7% females, with a male to female ratio of 1: 1.3. Fig 2 shows the age and sex distribution in the surveyed population.

10,571 households were registered for the study. The overall mean household size was 6.2. The use of improved sanitation facilities was low with 8.6% (95%CI: 6.7–10.8%), across all surveyed areas. The highest use of improved latrines was in Lawra EU where 29.8% of the population accessed an improved facility. Access to unimproved latrines was higher at 15.5% (95% CI: 12.7–18.9%) of households. The majority of households used no toilet facilities. Of the households with a latrine, 16.3% (95% CI: 11.9–22.1) had access to a hand-washing facility within 15 meters of the latrine. 7.9% (95% CI: 5.3–11.8% of households and 4.3% (95% CI: 2.6–7.0%) had water and soap available at the hand washing facility respectively. 75.9% (95%CI 71.5–79.8%, EU range 47.4–90.1%) of households had access to an improved water source (S2 Table), with 45.5% (95% CI 41.5–49.7, EU range 28.4–61.8%) making a round trip of water collection in < 30 minutes.

### Clinical examinations

#### Facial cleanliness

In terms of facial cleanliness, 5.1% of children aged 1–9 years had ocular discharge. 20.5% of children aged 1–9 years had nasal discharge.

#### Clinical assessments for trachoma

**Fig 3:**
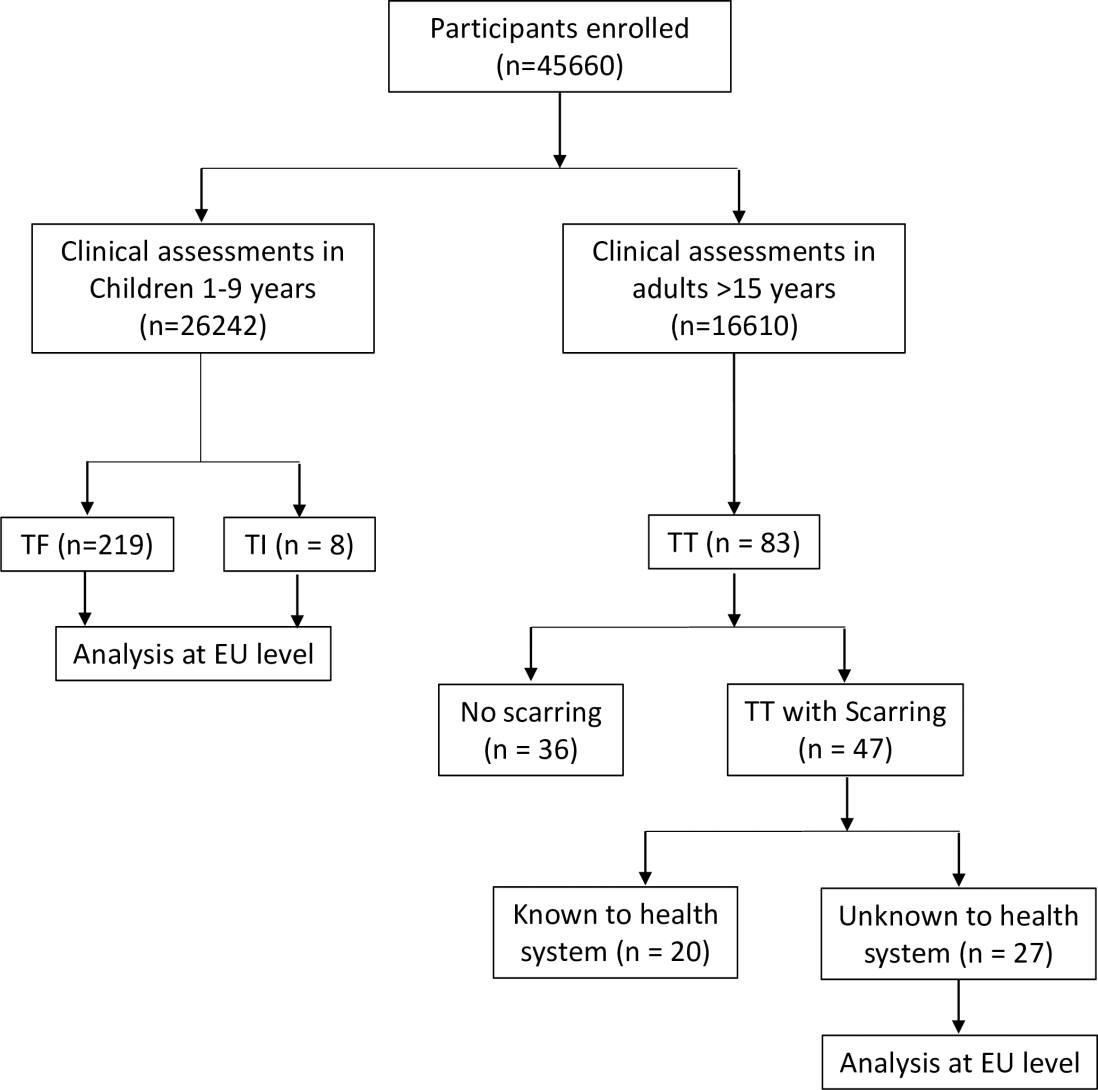
Presentation of number of active trachoma cases identified during the survey.

**Fig 4:**
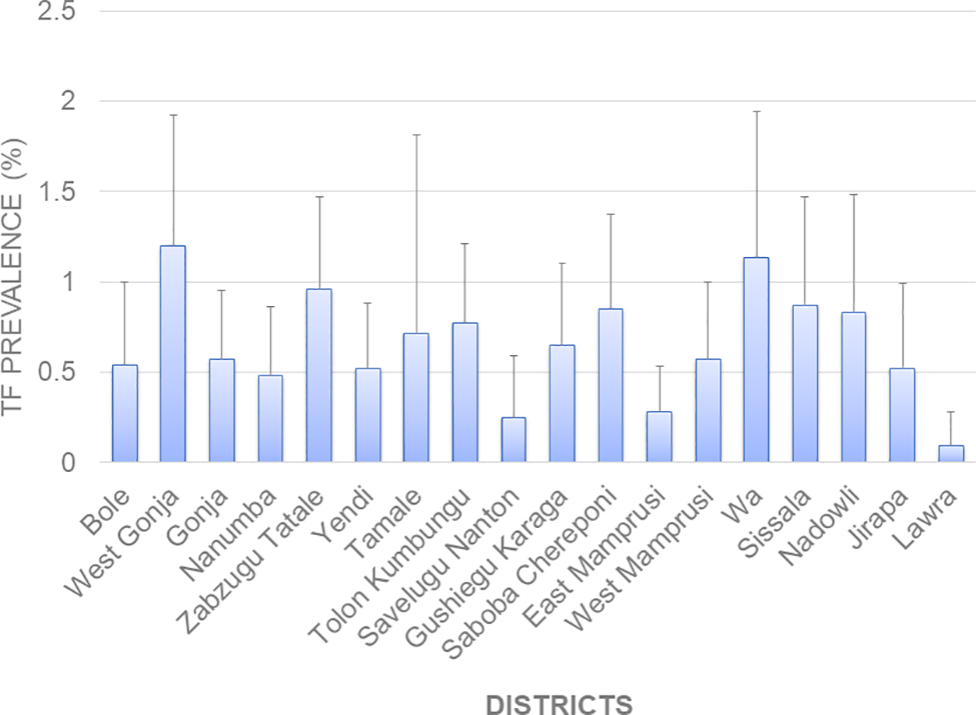
TF prevalence by survey district.

Fig 3 presents a breakdown of the cases identified, with the S3 Table presenting a breakdown of cases by EU. A total of 26,242 children aged 1–9 years old were examined for signs of TF and 219 children were positive upon examination. Most TF cases were in children 3 to 6 years (S1 Fig). The EU mean cluster TF prevalence ranged from 0.1% to 1.2%. Fig 4 shows the TF prevalence for each survey EU. In this study, 13 individuals between the ages of 10–72 years were also identified with TF. A total of 8 children were identified with TI. Of these, 7 (0.03%) children had unilateral TI, and 1 (0.004%) had bilateral TI.

**Fig 5:**
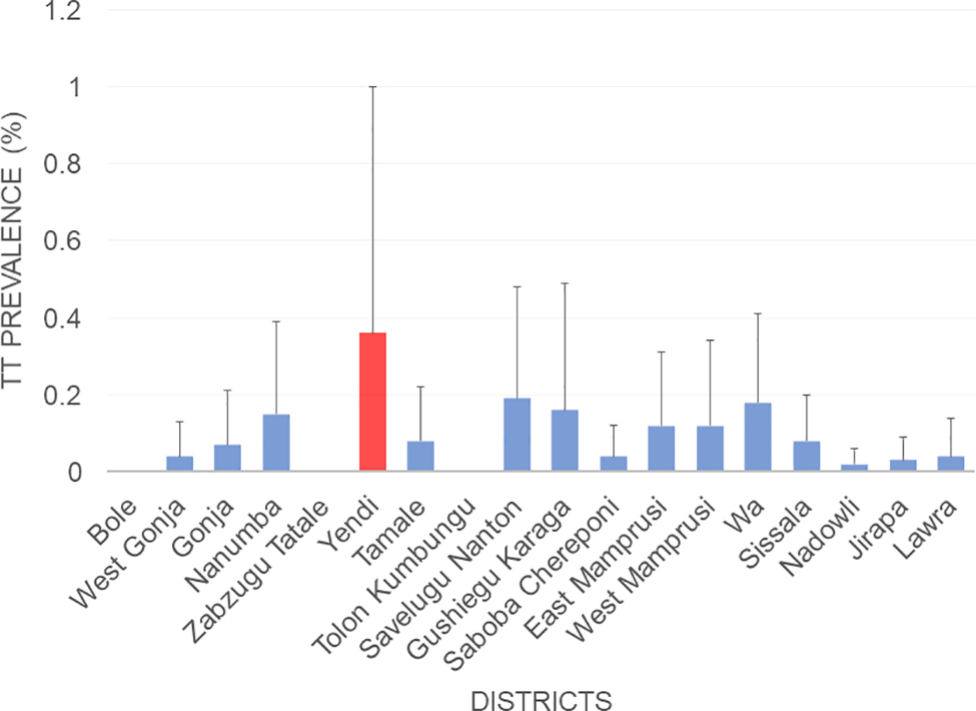
TT prevalence by survey district.

Overall, there were 83 TT cases in adults aged 15 and above. Of these, 47 had TS. TT cases were in individuals aged at least 40 years and above (42–100 years), with 70% of cases in people above 65 years. Of all TS cases, 27 were unknown to the health system. The EU prevalence of TT unknown to the health system ranged from 0.00 to 0.36%. Wa, Gushiegu Karaga and Savelugu Nanton and Yendi had the highest TT estimates of 0.18% (95% CI 0.0–0.41), 0.16% (95% CI: 0.00–0.49), 0.19% (95% CI: 0.00–0.48) and 0.36% (95% CI: 0.00–1.00) respectively [Fig 5]. Based on these results, Yendi is the only EU that had not reached the elimination threshold of < 0.2% TT prevalence in adults aged 15 and above.

**Fig 6:**
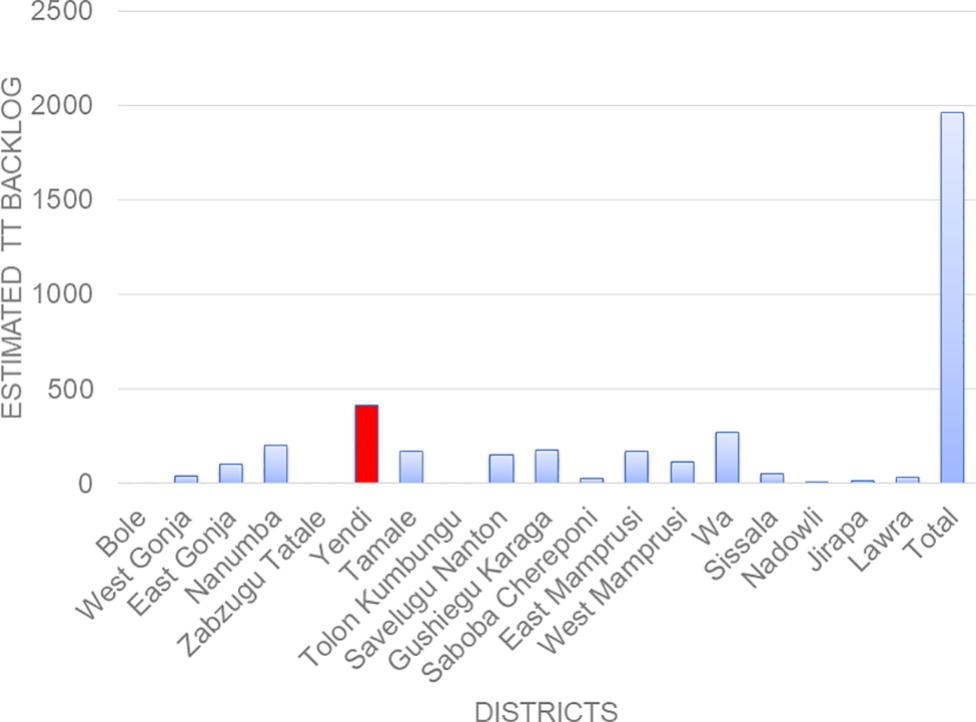
Estimated TT backlog in survey districts.

Based on these results a cumulative TT backlog of 1958 cases has been estimated for all the EUs (Fig 6). The estimated TT backlog for Yendi is 417. All TT patients identified in the study will be offered surgery.

## Discussions

The WHO Strategic and Technical Advisory Group (STAG) on NTDs recommends that sentinel site data are no longer recommended for trachoma elimination, but the validation is to be primarily based upon a single cross-sectional prevalence survey, to be conducted at the district level, two years after impact surveys indicate that elimination targets have been reached [4]. After a decade of programme activities, followed by at least four years of post-intervention surveillance activities, this study aimed at evaluating the prevalence of trachoma as a first step towards the validation of Ghana as trachoma-free country. With this survey, Ghana becomes one of the first countries to conduct a pre-validation survey at district level as per the new WHO guidelines [17].

This survey revealed the ability of the national programme in implementing the SAFE strategy to control trachoma. The results of TF prevalence show that the Ghana programme has sustained its achievement of reducing active trachoma (TF) in children aged 1–9 years to <5.0% in all 18 EUs within the previously endemic regions and has actually succeeded in reducing the prevalence further than previously reported [14]. There is therefore no evidence of resurgence of infection in Ghana. The very low prevalence of TF and TI suggests that very few incident cases of TT are to be expected in the future. The age-specific TF prevalence was highest in children 3–6 years, confirming results from other countries [27–30]. The findings also showed higher TF prevalence compared to TT, as observed in other settings [27].

TT cases were very low. The only EU that failed to meet the TT threshold is Yendi (0.36%). Most TT cases were observed in the older population, suggesting that there are few incident cases of TT in the population which may be expected in an elimination setting. There are some discussions over whether a population-based survey is the best methodology for estimating a rare occurrence such as TT cases in an elimination setting. This survey is not powered to be able to detect TT prevalence to a necessary precision and an alternative approach such as case-finding used in the guinea worm eradication programmes may be more effective in the endgame. Additionally, previous experience from the trachoma program has revealed that the number of TT cases identified following active TT case search is usually lower than the estimated cases from a survey. As such, it is possible that Yendi has actually reached the elimination threshold. Nonetheless, further evaluation will have to be undertaken in order to confirm these values, and the backlog of TT cases cleared via surgery. In the past TT surgery in Ghana proved problematic and appeared to be a persistent challenge to the program [14]. Thus, active TT case detection must continue and surgery undertaken through the support from the Ghana Health Service and its partners. All TT patients identified in the study, as well as through surveillance efforts will be offered surgery.

During the survey, it was observed that a number of adults were absent from the households. Community sensitization was not always well done and as such, the absent individuals are most likely healthy adults, away for their respective economic activities. This may be especially true for men who are the primary breadwinners in the surveyed regions. There is therefore the likelihood that the TT case prevalence reported may be overestimated. In terms of TF diagnosis, although all the graders were certified using GTMP methodologies and trainers, there remain difficulties in identifying follicles in the community and likely still some element of inter-observer dis-agreement. Further, TF is known to have poor specificity in relation to identifying *C*. *trachomatis* infection in low prevalence settings [31]. In regions with low levels of endemic trachoma, there is the possibility that much of the observed TF is attributable to non-chlamydial bacterial pathogens, as individuals who have previously developed a follicular conjunctivitis in response to *C*. *trachomatis* may more readily reform conjunctival follicles when challenged with certain other bacterial species [32]. As such, it is possible that the true *C*. *trachomatis* infection in the EUs may be lower than reported and alternative diagnostic indicators are needed to distinguish if transmission is on-going.

Ghana is close to becoming the first sub-Saharan country to eliminate trachoma as a public health problem. Ghana has often been a pace setter in the global trachoma community. It was in Ghana that the decision to carry out three rounds of antibiotics for hypo and meso endemic districts was taken. The current trend of carrying out house-to-house, community-by-community campaign for case search for TT patients was started by Ghana and the lessons have been shared with many endemic countries. Another lesson, which has been shared with the global community, is the Ghana national programme’s decision to counsel patients found with TT and offer immediate surgery that led to an improvement of TT surgical uptake. The key points of the Ghana programme include its strong leadership at all levels especially at the national level, the implementation of the full SAFE strategy right from the onset and great collaboration between the Ghana Health Service and all partners in the programme.

Despite the successes achieved over the past 15 years of programme implementation more effort is required, especially towards the sustainability of program gains and post-validation surveillance activities. Active TT case search in Yendi, followed by surgery to clear the backlog of cases would prepare the country in a position to complete and submit a dossier to WHO for validation of elimination of trachoma as a public health problem. This will need to be followed by post-validation surveillance, in order to ensure the gains made by the programme are sustained. As trachoma prevalence becomes very low, it is important to increase awareness and conduct systematic active TT case search, in order to mop up the remaining cases. This will require training ophthalmic nurses to conduct TT surgery for new cases identified during the post validation period. De-skilling of surgeons that are managing few and an ever-diminishing number of trichiasis cases will be an issue and how to adequately maintain quality of trichiasis surgeries is an important challenge that needs to be addressed in the endgame. Health workers and volunteers also need to be trained/ re-trained by ophthalmic nurses to heighten index of suspicion, to detect, register and refer TT cases for surgery. Establishing a reward system that will motivate community volunteers to identify and report cases for TT surgery could enhance a faster elimination and sustain the gains made. Such reward systems could be based on experience from previous programs such as smallpox [33] or guinea worm eradication [34] programs. Awareness for surgery using the mass media, print materials and community durbars must be enhanced. Increasing the acceptability of surgery will need to be intensified through health education sessions. The establishment of regional or district eye care centers also will promote routine eye examination, case search and TT surgeries. In addition to the above, water, sanitation and hygiene (WASH) may play a crucial role in the elimination of trachoma [35].

Other studies have shown Ghana has made a significant improvement in population access to improved water sources [36]. Improvement in access to water in rural areas has been better than in urban areas. Between 2003 and 2014 household access to improved water sources improved from 53.6% to 80.9% in rural Ghana while rural household access to latrines improved from 15.2% to 59.2% over the same period [19,37,38]. The F & E components of the SAFE strategy will have to be strengthened, in collaboration with other governmental and civil society organizations in order to sustain the gains in trachoma post-validation.

In conclusion, this survey has provided evidence that Ghana has nearly eliminated trachoma as a public health problem. Strategic plans will need to be developed to mop up the remaining TT cases in one outstanding EU, while an improvement in the F&E components of the SAFE strategy will firm up the gains made in eliminating trachoma in Ghana. Following an evaluation of Yendi, a dossier will be submitted to the WHO, to validate Ghana as having eliminated trachoma as a public health problem.

## Supporting information

S1 Checklist STROBE (STrengthening the Reporting of OBservational studies in Epidemiology) checklist. (DOC)

S1 Fig Prevalence of signs of active trachoma in children aged 1–9 years. (TIF)

S1 Table Trachoma prevalence in Northern and Upper West region districts at baseline and at follow-up. (DOCX)

S2 Table WASH indicators in the survey districts. (DOCX)

S3 Table Trachoma cases identified in each survey district. (DOCX)
