# Supplementary material for: Elimination of trachoma as a public health problem in Ghana: Providing evidence through a pre-validation survey
Source: PLoS Negl Trop Dis. 2017 Dec 12;11(12):e0006099. doi: 10.1371/journal.pntd.0006099 (PMC5746280; doi:10.1371/journal.pntd.0006099)
Supplement: S1 Checklist — (DOC) [file pntd.0006099.s001.doc]

STROBE Statement—Checklist of items that should be included in reports of ***cross-sectional studies***

|  | Item No | Recommendation | Section of manuscript |
| --- | --- | --- | --- |
| **Title and abstract** | 1 | (*a*) Indicate the study’s design with a commonly used term in the title or the abstract | Line 26 |
|  |  | (*b*) Provide in the abstract an informative and balanced summary of what was done and what was found | Lines 25-50 |
| Introduction | | |  |
| Background/rationale | 2 | Explain the scientific background and rationale for the investigation being reported | Lines 77-162 |
| Objectives | 3 | State specific objectives, including any prespecified hypotheses | Lines 160-162 |
| Methods | | |  |
| Study design | 4 | Present key elements of study design early in the paper | Lines 180-182 |
| Setting | 5 | Describe the setting, locations, and relevant dates, including periods of recruitment, exposure, follow-up, and data collection | Lines 183-229 |
| Participants | 6 | (*a*) Give the eligibility criteria, and the sources and methods of selection of participants | Lines 168-173, 215. |
| Variables | 7 | Clearly define all outcomes, exposures, predictors, potential confounders, and effect modifiers. Give diagnostic criteria, if applicable | Lines 227-235. |
| Data sources/ measurement | 8* | For each variable of interest, give sources of data and details of methods of assessment (measurement). Describe comparability of assessment methods if there is more than one group | Lines 227-235 |
| Bias | 9 | Describe any efforts to address potential sources of bias |  |
| Study size | 10 | Explain how the study size was arrived at | Lines 220-226 |
| Quantitative variables | 11 | Explain how quantitative variables were handled in the analyses. If applicable, describe which groupings were chosen and why | Lines 245-259 |
| Statistical methods | 12 | (*a*) Describe all statistical methods, including those used to control for confounding | Lines 245-259 |
|  |  | (*b*) Describe any methods used to examine subgroups and interactions | Lines 245-259 |
|  |  | (*c*) Explain how missing data were addressed | None |
|  |  | (*d*) If applicable, describe analytical methods taking account of sampling strategy | Lines 245-259 |
|  |  | (*e*) Describe any sensitivity analyses | None |
| Results | | |  |
| Participants | 13* | (a) Report numbers of individuals at each stage of study—eg numbers potentially eligible, examined for eligibility, confirmed eligible, included in the study, completing follow-up, and analysed | Lines 278-282 |
|  |  | (b) Give reasons for non-participation at each stage | None |
|  |  | (c) Consider use of a flow diagram | Figure 3 |
| Descriptive data | 14* | (a) Give characteristics of study participants (eg demographic, clinical, social) and information on exposures and potential confounders | Lines 278-290 |
|  |  | (b) Indicate number of participants with missing data for each variable of interest | Not applicable |
| Outcome data | 15* | Report numbers of outcome events or summary measures | Lines 294-323 |
| Main results | 16 | (*a*) Give unadjusted estimates and, if applicable, confounder-adjusted estimates and their precision (eg, 95% confidence interval). Make clear which confounders were adjusted for and why they were included | Lines 300-316 |
|  |  | (*b*) Report category boundaries when continuous variables were categorized |  |
|  |  | (*c*) If relevant, consider translating estimates of relative risk into absolute risk for a meaningful time period |  |
| Other analyses | 17 | Report other analyses done—eg analyses of subgroups and interactions, and sensitivity analyses | None |
| Discussion | | |  |
| Key results | 18 | Summarise key results with reference to study objectives | Lines 338-380 |
| Limitations | 19 | Discuss limitations of the study, taking into account sources of potential bias or imprecision. Discuss both direction and magnitude of any potential bias | Lines 382-398 |
| Interpretation | 20 | Give a cautious overall interpretation of results considering objectives, limitations, multiplicity of analyses, results from similar studies, and other relevant evidence | Lines 382-398 |
| Generalisability | 21 | Discuss the generalisability (external validity) of the study results | Lines 382-398 |
| Other information | | |  |
| Funding | 22 | Give the source of funding and the role of the funders for the present study and, if applicable, for the original study on which the present article is based | Information provided |

*Give information separately for exposed and unexposed groups.

**Note:** An Explanation and Elaboration article discusses each checklist item and gives methodological background and published examples of transparent reporting. The STROBE checklist is best used in conjunction with this article (freely available on the Web sites of PLoS Medicine at http://www.plosmedicine.org/, Annals of Internal Medicine at http://www.annals.org/, and Epidemiology at http://www.epidem.com/). Information on the STROBE Initiative is available at www.strobe-statement.org.
