# Supplementary figures and images for: Elimination of trachoma as a public health problem in Ghana: Providing evidence through a pre-validation survey
Source: PLoS Negl Trop Dis. 2017 Dec 12;11(12):e0006099. doi: 10.1371/journal.pntd.0006099 (PMC5746280; doi:10.1371/journal.pntd.0006099)

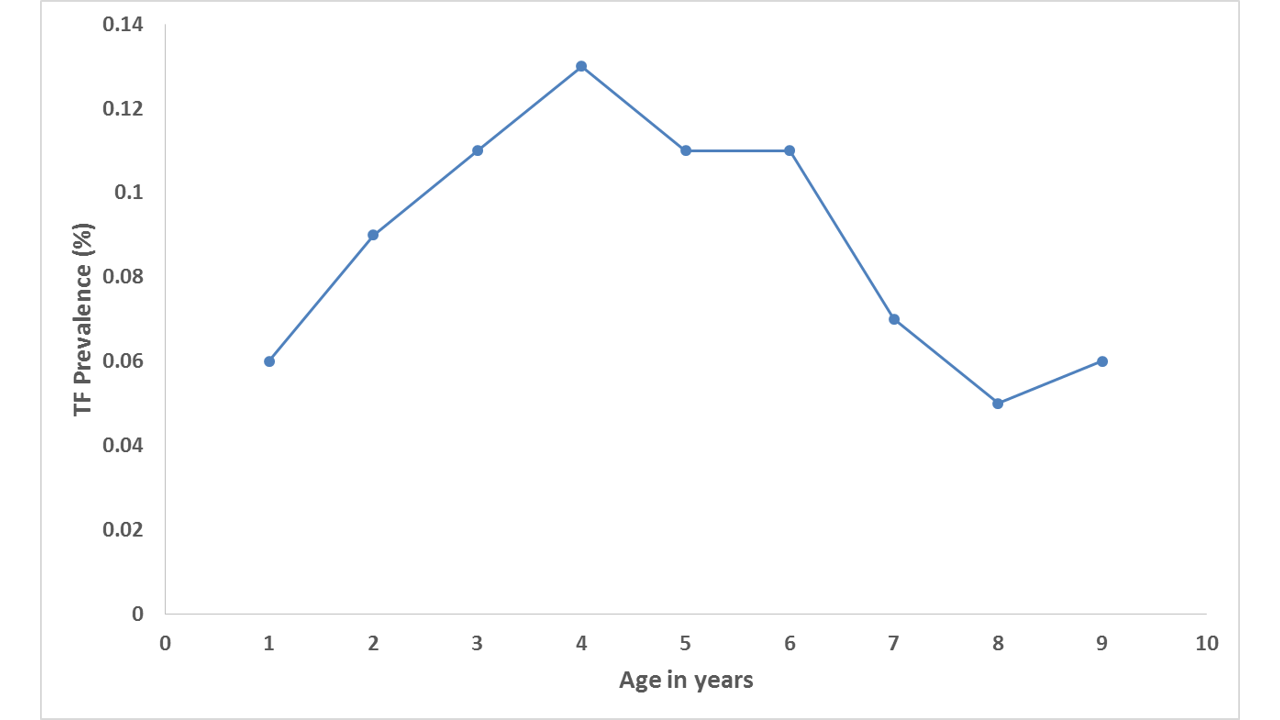

Supplement: S1 Fig — (TIF) [file pntd.0006099.s002.tif]
