# Supplementary material for: Elimination of trachoma as a public health problem in Ghana: Providing evidence through a pre-validation survey
Source: PLoS Negl Trop Dis. 2017 Dec 12;11(12):e0006099. doi: 10.1371/journal.pntd.0006099 (PMC5746280; doi:10.1371/journal.pntd.0006099)
Supplement: S1 Table — (DOCX) [file pntd.0006099.s003.docx]

Supplementary Table 1: Trachoma prevalence in Northern and Upper West region districts at baseline and at follow-up

|  | **Baseline (2000-2003)** | | | **Follow-up/Impact Assessment (2008)** | | |
| --- | --- | --- | --- | --- | --- | --- |
| **District** | **%TF** | **%TFTI** | **%TT** | **%TF** | **%TFTI** | **%TT** |
| Tolon Kumbungu | - | 12.4 | 8.4 | 0.19 | - | 0.33 |
| West Gonja | - | 11.7 | 3.7 | 0.14 | - | 0.76 |
| Sissala | - | 11.5 | 1.6 | 0.83 | 0.88 | 1.07 |
| Wa | - | 16.1 | 2.6 | 1.34 | - | 0.57 |
| Savelugu Nanton |  | 9.7 | 4.5 | 1.15 | - | 0.52 |
| Bole | 8.2 | - | 1.8 | 2.81 | 0.07 | 0.31 |
| West Mamprusi | 6.8 | - | 0.8 | 0.88 | - | 0.47 |
| Zabzugu/ Tatale | 6.7 | - | 0.4 | 1.66 | - | - |
| Tamale Municipal |  | 4.7 | 4.9 | 0.53 | - | 0.41 |
| Jirapa/ Lambussie | 5.0 | - | 0.8 | 0.57 | - | 0.34 |
| Gushiegu/ Karaga | 4.4 | - | 0.8 | 0.97 | - | 0.19 |
| Nanumba | 3.8 | - | 0.5 | 1.88 | 0.12 | 0.05 |
| Gonja | 3.7 | - | 0.9 | 0.38 | - | 0.09 |
| Nadowli | 3.6 | - | 1.3 | 0.15 | - | 0.19 |
| Yendi | 3.5 | - | 1 | 0.31 | - | 0.23 |
| Saboba/ Chereponi | 3.2 | - | 0.5 | 0.38 | - | - |
| Lawra | 2.8 | - | 0.7 | 0.18 | 0.26 | 0.15 |
| East Mamprusi | 2.8 | - | 0.6 | 0.36 | - | 0.13 |
