# Supplementary material for: Elimination of trachoma as a public health problem in Ghana: Providing evidence through a pre-validation survey
Source: PLoS Negl Trop Dis. 2017 Dec 12;11(12):e0006099. doi: 10.1371/journal.pntd.0006099 (PMC5746280; doi:10.1371/journal.pntd.0006099)
Supplement: S2 Table — (DOCX) [file pntd.0006099.s004.docx]

|  | **Indicators** | **Percentage (%)** | **95% CI Lower bound** | **95% CI**  **Upper bound** |
| --- | --- | --- | --- | --- |
| Drinking water | Improved drinking water | 75.9 | 71.5 | 79.8 |
|  | Unimproved drinking water | 2.6 | 1.6 | 4.3 |
|  | Surface drinking water | 21.5 | 17.8 | 25.8 |
|  | Drinking water in yard | 1.4 | 0.8 | 2.4 |
|  | Drinking water < 30 min | 39.6 | 35.6 | 43.6 |
|  | Drinking water > 30 min | 42.5 | 38.7 | 46.4 |
|  | Drinking water > 1 hr | 16.5 | 13.6 | 20.0 |
| Washing water | Improved washing water | 75.6 | 71.2 | 79.5 |
|  | Unimproved washing water | 2.7 | 1.6 | 4.4 |
|  | Surface washing water | 21.7 | 17.9 | 26.0 |
|  | Washing water at source | 0.0 | 0.0 | 0.1 |
|  | Washing water < 30 min | 45.5 | 41.5 | 49.7 |
|  | Washing water > 30 min | 54.5 | 50.3 | 58.5 |
| Toilet facilities | Shared latrine | 13.0 | 10.4 | 16.2 |
|  | Private latrine | 10.9 | 8.8 | 13.4 |
|  | Near house | 0.6 | 0.5 | 13.4 |
|  | Bush | 75.5 | 71.7 | 78.9 |
|  | other | 0.0 | 0.0 | 0.1 |
|  | Improved latrine | 8.6 | 6.7 | 10.8 |
|  | Unimproved latrine | 15.5 | 12.7 | 18.9 |
|  | No toilet | 75.9 | 72.1 | 79.3 |
| Soap and water at toilet facility | Water for hand washing | 8.0 | 5.3 | 11.8 |
|  | No water for hand washing | 91.8 | 88.0 | 94.5 |
|  | Other | 0.2 | 0.1 | 0.6 |
|  | Soap available | 4.3 | 2.6 | 7.0 |
|  | No soap | 95.4 | 92.6 | 97.2 |
|  | Other | 0.3 | 0.1 | 0.6 |

Supplementary Table 2: Water and Sanitation Indicators in the study districts
