# Supplementary material for: Elimination of trachoma as a public health problem in Ghana: Providing evidence through a pre-validation survey
Source: PLoS Negl Trop Dis. 2017 Dec 12;11(12):e0006099. doi: 10.1371/journal.pntd.0006099 (PMC5746280; doi:10.1371/journal.pntd.0006099)
Supplement: S3 Table — (DOCX) [file pntd.0006099.s005.docx]

Supplementary Table 3: Trachoma cases identified in each survey district

| **District** | **Number Examined** | **1-9 years** | **15 years up** | **TF** | **TT only** | **TT with TS** | **TT_unknown** |
| --- | --- | --- | --- | --- | --- | --- | --- |
| Bole | 2162 | 1333 | 669 | 9 | **1** | **0** | **0** |
| West Gonja | 1811 | 1311 | 401 | 25 | **4** | **1** | **1** |
| Gonja | 2688 | 1325 | 1093 | 11 | **3** | **1** | **1** |
| Nanumba | 3571 | 2119 | 1150 | 20 | **6** | **3** | **2** |
| Zabzugu Tatale | 2401 | 1656 | 611 | 35 | **1** | **0** | **0** |
| Yendi | 3103 | 1811 | 1063 | 13 | **5** | **5** | **5** |
| Tamale | 2608 | 1204 | 1208 | 4 | **3** | **2** | **2** |
| Tolon Kumbungu | 1924 | 1431 | 449 | 15 | **0** | **0** | **0** |
| Savelugu Nanton | 2656 | 1390 | 1113 | 4 | **5** | **2** | **2** |
| Gushiegu Karaga | 1947 | 1388 | 426 | 16 | **2** | **1** | **1** |
| Saboba Chereponi | 2096 | 1342 | 588 | 11 | **1** | **1** | **1** |
| East Mamprusi | 2998 | 1654 | 1059 | 6 | **2** | **2** | **2** |
| West Mamprusi | 3156 | 1794 | 1047 | 13 | **8** | **4** | **2** |
| Wa | 2869 | 1395 | 1254 | 19 | **12** | **8** | **2** |
| Sissala | 2997 | 1426 | 1304 | 15 | **10** | **7** | **2** |
| Nadowli | 2322 | 1521 | 720 | 18 | **19** | **4** | **2** |
| Jirapa | 2222 | 1381 | 722 | 10 | **7** | **3** | **1** |
| Lawra | 2129 | 761 | 1196 | 1 | **4** | **2** | **1** |
